# Correlation of Solidification Thermal Variables with Microstructure and Hardness in CuMn11Al8Fe3Ni3 Manganese–Aluminum–Bronze Alloy

**DOI:** 10.3390/ma18020234

**Published:** 2025-01-08

**Authors:** Ricardo de Luca, Paulo Henrique Tedardi do Nascimento, Vinicius Torres dos Santos, Marcio Rodrigues da Silva, Flavia Gonçalves Lobo, Rogerio Teram, Mauricio Silva Nascimento, Antonio Augusto Couto, Anibal de Andrade Mendes Filho, Givanildo Alves dos Santos

**Affiliations:** 1 Termomecanica São Paulo S.A., São Bernardo do Campo 09612-000, Brazil; ricardo.luca@termomecanica.com.br (R.d.L.); vinicius.santos@termomecanica.com.br (V.T.d.S.); marcio.rodrigues@termomecanica.com.br (M.R.d.S.); 2 Department of Engineering, Mackenzie Presbyterian University, São Paulo 01302-907, Brazil; antonioaugusto.couto@mackenzie.br; 3 Department of Mechanics, Federal Institute of Education, Science and Technology of São Paulo, São Paulo 01109-010, Brazil; rogerioteram@ifsp.edu.br (R.T.); mauricio.nascimento@ifsp.edu.br (M.S.N.); givanildo@ifsp.edu.br (G.A.d.S.); 4 Department of Engineering, Modeling and Applied Social Sciences, Federal University of ABC, Santo André 09210-580, Brazil; anibal.mendes@ufabc.edu.br

**Keywords:** manganese–aluminum–bronze, solidification thermal variables, unidirectional solidification, microstructure

## Abstract

The mechanical properties of a final product are directly influenced by the solidification process, chemical composition heterogeneity, and the thermal variables during solidification. This study aims to analyze the influence of solidification thermal variables on the microstructure, hardness, and phase distribution of the CuMn11Al8Fe3Ni3. The alloy was directionally and upward solidified from a temperature of 1250 °C. Heat extraction occurred through a water-cooled AISI 1020 steel interface. The thermal variables were recorded using a data acquisition system, with temperature monitored at seven different positions, where cooling rates varied from 13.03 °C/s at the closest position to 0.23 °C/s at the farthest. The Brinell hardness decreased from 199 HB at the highest cooling rate position to 184 HB at the slowest cooling rate position. This indicates that higher cooling rates increase the hardness of the alloy, which can be attributed to the stabilization of the metastable β phase with refined and equiaxial grains due to iron addition. Vickers microhardness was observed in regions subjected to slower cooling (244 HV) compared to faster cooling regions (222 HV). Therefore, the correlation between solidification thermal variables and alloy properties provides valuable insights into the relationship between microstructure and the properties of the CuMn11Al8Fe3Ni3 alloy.

## 1. Introduction

Aluminum–bronze alloys are alloys where the main element is copper and the main addition element of the alloy is aluminum, and may contain additions of silicon, manganese, iron, and nickel as minor alloy elements to create different types of alloys with properties designed to meet different requirements, such as mechanical strength, ductility, corrosion resistance, magnetic permeability, and wear resistance. The first productions were recorded in the 1850s; initially, the production consisted of binary alloys of 6–11% aluminum and then other elements were added in appropriate proportions in search of differentiated properties [1,2].

The addition of aluminum bronzes reduces the density of the alloy because aluminum is 29% the density of copper; it also provides increased mechanical strength, wear resistance, and corrosion resistance. The addition of Fe to the alloy causes the grain size to be refined, increasing the impact resistance and mechanical strength. Due to the complete solubility of Ni in copper, it is commonly associated with iron in this alloy to provide mechanical strength, hardness, and corrosion resistance. The addition of Mn improves the casting quality in the mold where this alloy is mainly applied and improves the corrosion resistance and increases the mechanical strength [3,4].

Manganese–aluminum–bronze (MAB) alloys, classified as part of the “High Strength” group within the aluminum bronze family, are increasingly employed in the naval and defense industries due to their superior mechanical properties compared to other copper alloys. Specifically, the CuMn11Al8Fe3Ni3 manganese–aluminum–bronze alloy is recognized for its excellent corrosion resistance in various environments, particularly marine conditions, as well as its notable tribological properties, including high wear resistance and resistance to erosion and cavitation [5,6,7]. These attributes make this alloy highly versatile for use in components such as valves, bushings, bearings, turbines, and marine propellers [8,9,10,11].

**Figure 1:**
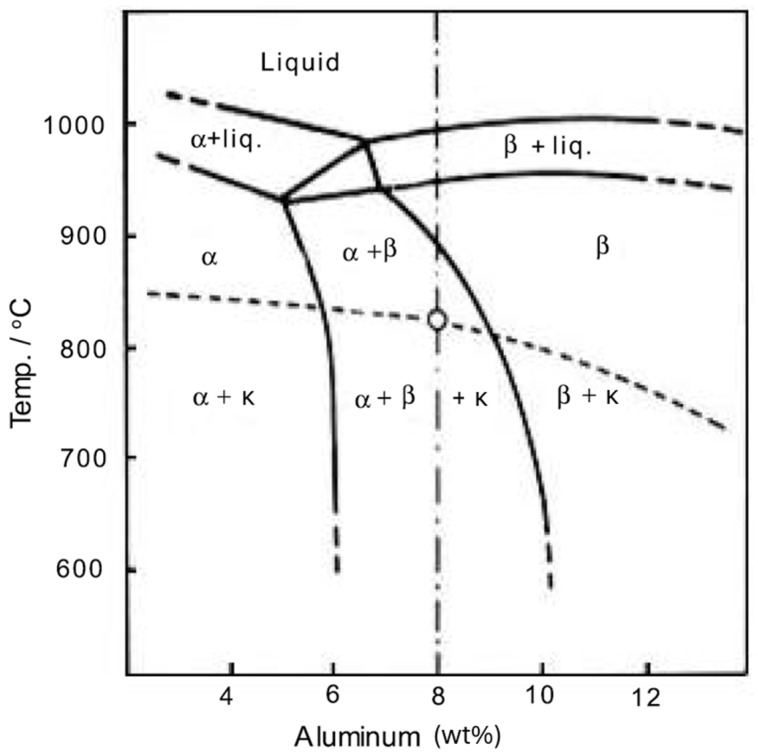
Pseudo-binary diagram of Cu-Al-11Mn [12].

The phase transformations evolution expected for the CuMn11Al8Fe3Ni3 alloy is shown in Figure 1. Additionally, this alloy is expected to exhibit a microstructure very similar to that of NAB (nickel–aluminum–bronze) with the same aluminum content, as studied by [8,12], which consists of FCC α-phase grains and elongated BCC β-phase precipitates in large or small dendrite particles and globular and cuboid particles [13].

The microstructure is expected to primarily consist of α and β phases, with κ intermetallic phase precipitates. The presence of Mn, along with small amounts of Fe and Ni, minimally affects the phase boundary positions but promotes the precipitation of the κ intermetallic phase within the α and β phases, particularly in the temperature range of 750–850 °C. Optical micrographs revealed α as the lighter phase, β as the darker phase, and the κ intermetallic phase with cuboid, globular, or dendritic morphologies distributed within the α and β phase domains [8,12]. Under typical conditions, slow cooling of this alloy does not adversely affect its structure or mechanical properties. However, faster cooling rates could enhance productivity and lead to variations in microstructure and potentially unexpected mechanical behavior. Changes in hardness are primarily associated with the atomic rearrangement of the β phase, which, as previously noted, significantly influences the mechanical properties [14,15,16]. The typical chemical composition of these precipitation forms is shown in Table 1.

Due to the complex structure of MAB, intermetallics and their use in specific applications such as the naval industry, research relating to the use of the main aluminum bronze alloys (MAB and SAB) in severe applications has become common [17,18]. Large areas of common research have been applied to studies regarding the verification of corrosion performance [6,19,20], chemical composition [21,22], additive manufacturing [23,24,25,26], heat treatment [7,27,28,29], machining [30] and surface treatment [19], and microstructural control [21,31,32]. Due to the large areas of research, the versatility of uses of aluminum–bronze alloys is demonstrated and such applications characterize the high performance of these alloys and demonstrate a large field for development and studies of scientific methodology.

This study then investigates the influence of solidification thermal variables on the microhardness, hardness, and microstructure of the CuMn11Al8Fe3Ni3 manganese–aluminum–bronze alloy, highlighting the crucial role of cooling rate in the alloy’s solidification process. The investigation was made following an upward unidirectional solidification process, and different microstructures were observed along the length of the CuMn11Al8Fe3Ni3 alloy ingot, which significantly affected its properties. This study is essential for understanding how cooling rates influence the hardness and microstructure of the alloy. The equations derived in this work are highly relevant for industrial applications, as they enable the correlation between the final microstructure and the cooling rate. For instance, applications requiring higher Brinell macrohardness with fewer precipitates can use these findings to optimize solidification parameters and achieve the desired material properties. Directional solidification enables the formation of distinct microstructures within the same ingot, thereby influencing the material’s overall performance. Numerous studies have highlighted the effects of manufacturing and thermomechanical processes on the microstructure and properties of engineering materials. In this research, the solidification thermal parameters, such as tip growth rate (V_L_) and cooling rate (T_R_), were correlated with hardness and microhardness. Microstructural analysis was performed using optical microscopy and scanning electron microscopy (SEM) at various positions along the ingot to assess the evolution of the microstructure. To the best of our knowledge, no previous studies have specifically investigated the CuMn11Al8Fe3Ni3 alloy following upward unidirectional solidification in the existing scientific literature [33,34].

## 2. Materials and Methods

The CuMn11Al8Fe3Ni3 alloy was supplied by Termomecanica São Paulo S.A. (São Bernardo do Campo, Brazil) in the form of 66.5 mm diameter cylindrical bars. The chemical composition of the supplied bars is presented in Table 2.

### 2.1. Unidirectional Solidification

**Figure 2:**
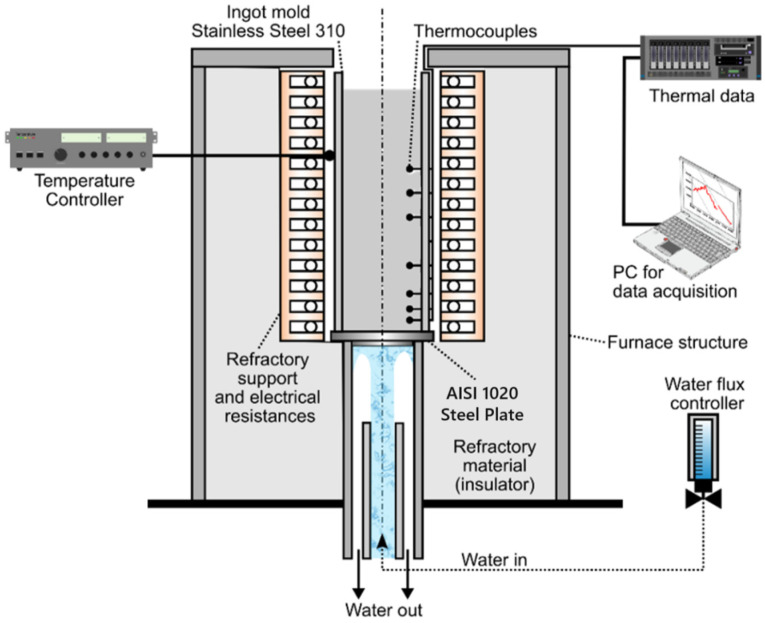
Schematic representation of the unidirectional solidification apparatus used to measure the thermal variables of the CuMn11Al8Fe3Ni3 alloy [13,33].

The furnace was constructed with a metallic frame, internally lined with refractory material, featuring a circular shape and an opening at the top for inserting the ingot mold. Electrical heating elements were attached inside the furnace to heat the material. The cooling pipes enter through a hole in the lower part of the furnace and are routed through thermally insulated tubing. The exit point of the cooling system is located at the bottom of the ingot mold. As a result, when water is activated, both the ingot mold and the material undergo upward unidirectional cooling. After absorbing heat, the water flows downward through the gap between the cooling pipes and the insulated tubing, eventually exiting at the furnace’s lower section. The ingot mold, made of AISI 304 stainless steel, has an internal diameter of 65.00 mm and a height of 120 mm. It has 7 holes with a diameter of 2 mm along its height in order to allow the thermocouples to enter it. Temperature measurements were recorded using K-type thermocouples from the Exacta brand, with solid wire insulated with ceramic fiber with resolution ±1 °C. Positioned at distances of 4, 8, 12, 16, 26, 35, and 53 mm from the cooling base, and inserted approximately 30 mm into the 65 mm diameter ingot mold [35,36,37]. These thermocouples, which were thin and uncoated, reached thermal equilibrium with the molten alloy prior to the activation of the cooling water at the base of the solidification apparatus. Their small size and high sampling frequency allowed for accurate temperature readings, particularly in regions with higher cooling rates. Then, the set was added to the unidirectional solidification furnace, and the ingot mold’s thermocouples were connected to an acquisition system formed of an NI 9212 module from National Instruments (National Instruments, Debrecen, Hungary) and an NI cDAQ 9171 chassis from the same company (National Instruments, Debrecen, Hungary). Initially, about 2 kg of CuMn11Al8Fe3Ni3 alloy was melted in a Salamander SIC AS2 graphite crucible using a muffle furnace heated to 1250 °C, which is above the alloy’s liquidus temperature of 990 °C, and held for 10 min to ensure temperature uniformity. Subsequently, the molten alloy was poured into the ingot mold, preheated to 1100 °C, within the unidirectional solidification apparatus. The solidification began when a water jet, flowing at 18 L/min, was initiated to cool the base of the apparatus. Temperature and time data from each thermocouple during cooling were read and recorded using NI LabView Signal Express data acquisition software version 14.0.0, installed on a computer connected to the acquisition system is presented in Figure 2.

It is important to note that the results of this study are based on a single unidirectional solidification experiment. While this approach allows for precise control and analysis of thermal variables and their effects on microstructure and hardness, it may not capture potential variations that could arise from repeated experiments.

### 2.2. Thermal Variables and Methods

The correlation between the position (P) and the time of passage of the liquidus isotherm (t_L_) can be represented according to P = C(t_L_)^n^; the methodologies for determining the casting thermal parameters were the same as those used in the studies [13,33,38].

The tip growth rate (VL) was determined by analyzing the function P = f(t), which defines the relationship between the position of the thermocouple (P) and the time elapsed from the start of alloy cooling to the moment the liquidus temperature (t_L_) is detected by each thermocouple. In this context, VL represents the rate at which the solidification front advances past each thermocouple.

The cooling rate (TR) at each thermocouple location was measured experimentally by assessing the temperature variation (ΔT) over a specified time interval (Δt) before and after the liquidus temperature was reached: T_R_ = ΔT/Δt. For this study, a temperature range of ΔT = ±5 °C around each thermocouple position P was used to calculate the cooling rate. Finally, the thermal gradient (GL) was computed using the formula GL = T_R_/V_L_ for each thermocouple position (P).

Microstructure characterization was carried out using optical microscopy (OM) and scanning electron microscopy (SEM). Metallographic analysis was performed on cross-sectional samples of the solidified ingot, taken from various distances (P) relative to the heat exchange surface, specifically at 4, 8, 12, 16, 26, 35, and 53 mm. SEM analysis, conducted using JSM-6010LA equipment (JEOL, Tokyo, Japan), examined the morphology of phases and intermetallics.

The mechanical properties were evaluated using Brinell hardness testing in accordance with ASTM E10 [39], with a 62.5 kgf load and a 2.5 mm diameter indenter. Hardness measurements were taken at five points along each thermocouple position. Vickers microhardness was also assessed following ASTM E92 [40] standards, using a 1 kgf load and a 15 s dwell time, at five different points along each thermocouple position (P).

Empirical power law relationships were established using Origin 10.0 software to model the dependence of thermal variables and hardness on position (P). These equations provide a useful tool for process design and for predicting hardness and microstructure outcomes.

### 2.3. Samples Preparation

**Figure 3:**
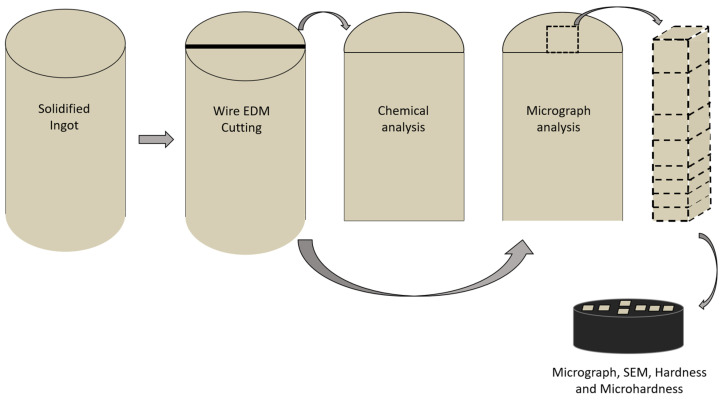
Schematic sequence used for sample cut and preparation.

The specimen preparation followed a similar procedure to that applied in our previous work [13,33]. The ingot was cut into two parts using wire electrical discharge machining (EDM). The first half of the material was subjected to chemical composition analysis using an X-ray fluorescence spectrometer to determine the chemical composition. Following this, the same section underwent macrographic analysis. The flat surface of the ingot was sequentially sanded with 220, 400, and 600 mesh grit water sandpaper, and then chemically etched using a solution composed of 50% HNO_3_ (nitric acid) and 50% water for approximately 7 s, following the parameters defined by solution E38 of the ASTM E407 standard [41], as shown in Figure 3.

For the second half of the ingot, samples were extracted again using wire electrical discharge machining (EDM), removing the central longitudinal section of the solidified ingot with a cross-sectional area of 1 cm^2^ and an approximate length of 100 mm. These samples were then cut transversely according to the thermocouple positions.

Next, the specimens were embedded in bakelite and numbered sequentially from 1 to 7, based on the thermocouple positions. These bakelite-mounted specimens were subjected to hardness and microhardness testing. After, they were also prepared for microstructural analysis, the samples were first sanded using water sandpaper with grit sizes of 220, 400, and 600, then polished with chromium oxide-based (5 μm) and alumina-based (1 μm) polishing rods. For chemical etching, the samples were immersed in a solution consisting of 5 g of FeCl_3_, 60 mL of ethanol, and 16 mL of HCl (following ASTM E407 solution 40 [41]) for 30 s. Afterward, the samples were rinsed with water and 70% ethanol. The microstructure was examined using optical microscopy and scanning electron microscopy.

## 3. Results

The composition of the ingot following unidirectional solidification is shown in Table 3. A comparison with the reference standard confirms that the composition remains within the limits of the DIN 1982 [42] specification. This analysis indicates that no contamination occurred during the solidification process, and there was no loss of alloying elements, ensuring the material continues to meet the required specifications of the standard.

### 3.1. Solidification Thermal Variables

**Figure 4:**
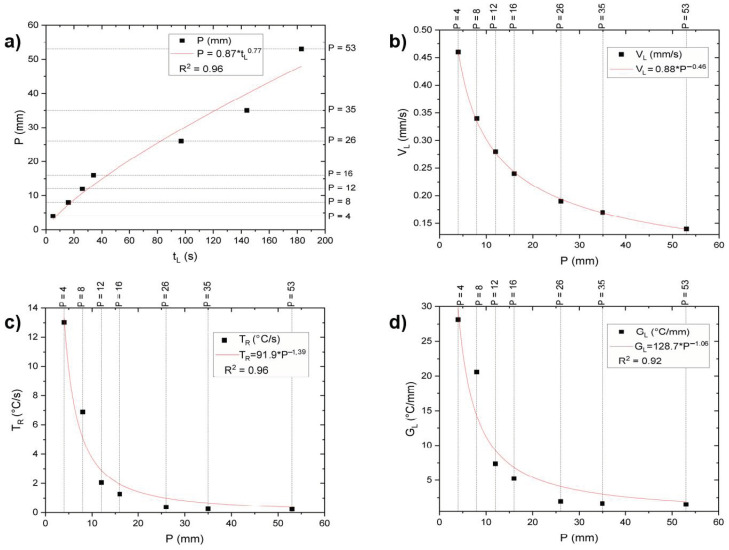
CuMn11Al8Fe3Ni3 during directional solidification. In (**a**), liquidus temperature (t_L_) is observed in each thermocouple; in (**b**), growth rate (V_L_) is evaluated for each thermocouple; in (**c**), the cooling rate (T_R_) is evaluated for each thermocouple; and in (**d**), the thermal gradient (G_L_) is evaluated for each thermocouple.

Figure 4 illustrates the thermal parameters t_L_, V_L_, T_R_, and G_L_, experimentally obtained as a function of the distance from the heat exchange surface (P). Power functions were fitted to each parameter, with an R-squared value greater than 0.9. As expected, all thermal parameters decreased with increasing P values during the directional solidification, as shown in Figure 4a–d. This decline can be attributed to the progressively increasing thermal resistance as solidification advances vertically upward, from the cooled base toward the top [4]. This reduction in thermal parameters inversely affects microstructural features, such as grain and precipitate size, which are discussed in subsequent sections.

### 3.2. Hardness and Microhardness

**Figure 5:**
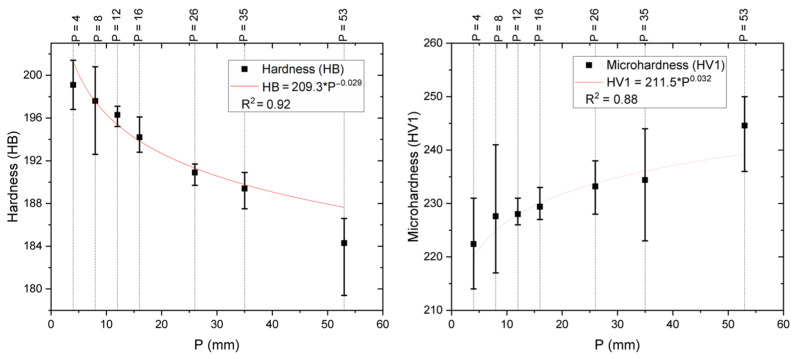
(**left**) Brinell hardness vs. position. (**right**) Vickers microhardness HV1 vs. position.

The measurements for hardness and microhardness are presented in Figure 5. The average macrohardness values (HB) decrease as the distance from the heat extraction surface increases. Conversely, the average microhardness values (HV) exhibit the opposite trend. The inverse relationship between hardness and microhardness can be explained by the measurement scale and the microstructural constituents involved. Brinell hardness is a macroscopic assessment technique, where the indentation is made over a relatively large area (approximately 2 mm), providing an average measure of a broad region composed of α and β phases, along with contributions from small precipitates. Consequently, the decrease in Brinell hardness with increasing distance from the heat extraction surface can be attributed to the presence of a coarser matrix microstructure, which is characterized by lower hardness and is typical of areas with slower cooling rates. In contrast, microhardness measurements reflect a local hardness at the indentation point (approximately 100 µm), and the observed trend is influenced by the increased volumetric fraction of precipitates, which results from slower cooling rates. This explanation is consistent with findings in references [13,33].

### 3.3. Phase Analysis

**Figure 6:**
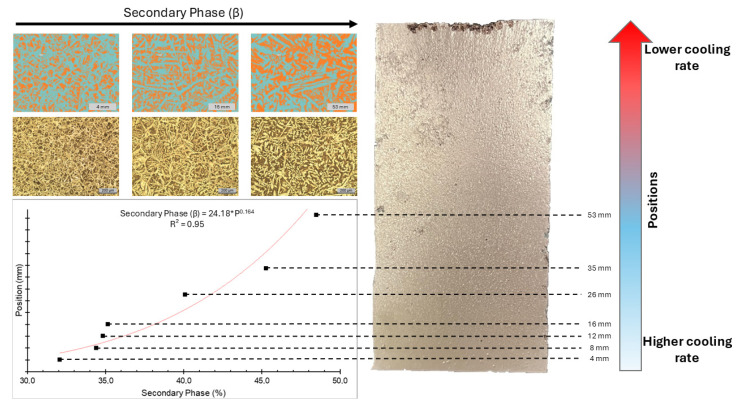
Correlation between the fraction of precipitates, the grain size, the shape of the precipitates, and the distance from the heat extraction surface.

At the first position (4 mm), the volume fraction of the secondary phase (β) is 32.10%, with the highest cooling rate among all positions (13.03 °C/s). At the second position (8 mm), where the cooling rate is lower (6.90 °C/s), the volume fraction increased to 34.40%. At the third position (12 mm), with a cooling rate of 2.05 °C/s, the volume fraction reached 34.80%. The fourth position (16 mm) experienced a cooling rate of 1.27 °C/s, resulting in a volume fraction of 35.20%. At the fifth position (26 mm), a cooling rate of 0.38 °C/s led to a secondary phase volume of 40.10%. The sixth position (35 mm), with a cooling rate of 0.25 °C/s, exhibited a volume fraction of 45.30%. Finally, at the seventh and farthest position (53 mm), the lowest cooling rate of 0.23 °C/s was recorded, producing the highest volume fraction of 48.50%. All positions, correlations between the secondary phase and position, macrography, and the distance from the cooling base are identified in Figure 6.

**Figure 7:**
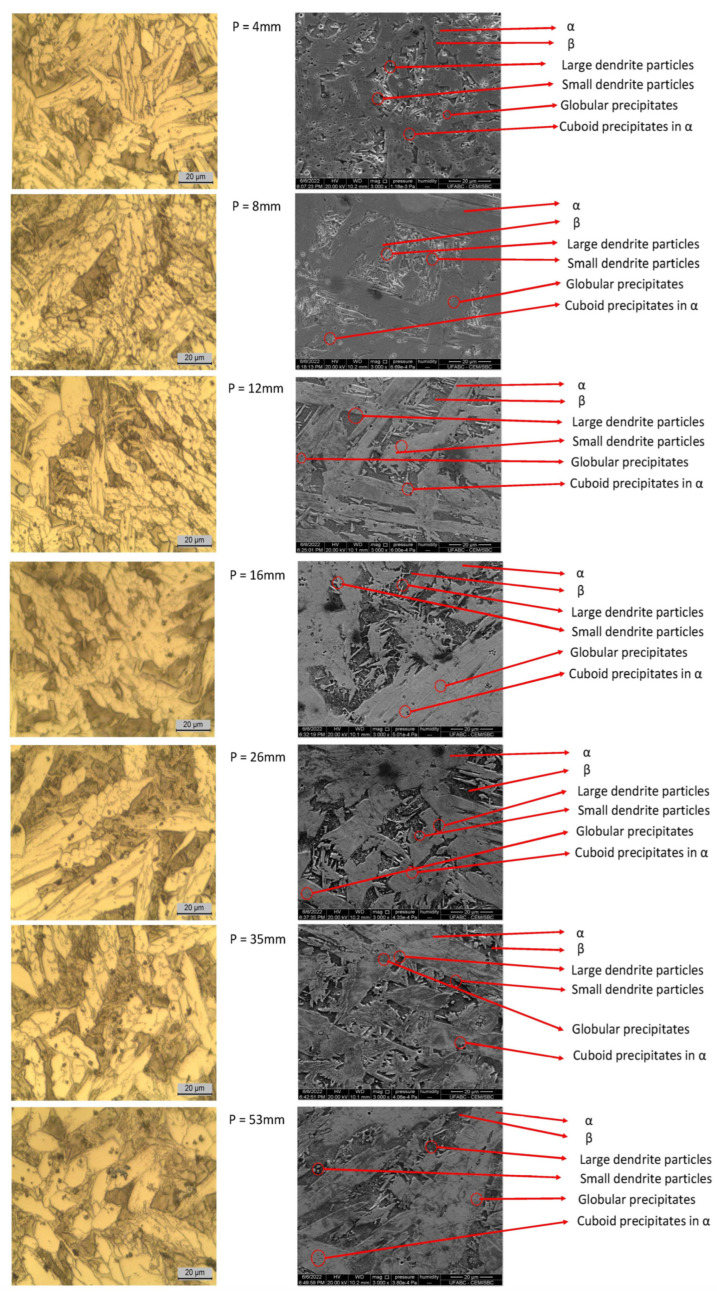
Optical microstructural analysis and phase identification via SE-SEM.

Optical and SEM micrographs are presented in Figure 7. Based on the micrographs (on the left-hand side), a clear microstructural evolution is observed with increasing distance from the cooling base and the corresponding diffusion time. In the SEM analysis (on the right-hand side), the microstructure was characterized according to the phase morphologies listed in Table 1. At the first position (4 mm), the microstructure showed a significant presence of α and β phases, along with intermetallics, likely in the form of large and small dendritic particles, and a minor appearance of globular and cuboid precipitates. This position had the lowest frequency of intermetallics and the lowest microhardness (222 HV), in contrast to its highest Brinell hardness value (199 HB), attributed to the predominant β phase.

In the subsequent positions, there was a noticeable increase in the β phase compared to the first position, and a gradual appearance of large and small dendritic intermetallic particles, as well as more globular and cuboid precipitates. At the 53 mm position, the microstructure showed a significant presence of α and β phases. According to [12], there was a possible decrease in the β phase and an increase in the α phase, with precipitates retained within the α phase, as confirmed by Brinell hardness measurements and secondary phase analysis [38].

The 53 mm position also had the highest frequency of intermetallics, with large and small dendritic particles, as well as globular and cuboid precipitates. This increase in precipitates was associated with higher Vickers microhardness, consistent with findings from [13,33,34,43]. Notably, this position recorded the highest microhardness value (244 HV).

The solidification thermal variables are presented in Table 4. Additionally, the hardness, microhardness, and the volume fraction of the secondary phase, measured at each respective position, are also provided.

## 4. Discussion

The results obtained from upward unidirectional solidification and the analysis of solidification thermal variables revealed that the highest liquidus isotherm displacement rates occurred near the ABNT 1020 steel base. These regions exhibited higher cooling rates, which contributed to greater hardness values. In contrast, the volume fraction of the secondary phase increased at positions farther from the base, where cooling rates were slower.

Microhardness measurements near the cooling base were lower than those recorded at greater distances. This behavior is attributed to the precipitation of intermetallic phases, as shown in Table 1 and Figure 5. The reduction in the copper-rich α phase near the base is directly associated with increased microhardness due to the higher concentration of intermetallic precipitates, a trend consistent with findings in other aluminum–bronze alloys.

The microhardness exhibited an opposite trend to hardness. Intermetallic phases, such as small dendritic particles and globular precipitates, became more prominent at positions farther from the interface. These intermetallics, which are rich in iron, explain the observed increase in microhardness at greater distances. Since these phases typically precipitate at lower temperatures, their prevalence is more pronounced in regions with slower cooling rates and extended diffusion times.

The increase in Vickers microhardness at farther positions can be attributed to the formation of a higher density and variety of intermetallics as the cooling rate decreases. These phases contribute significantly to the local hardness, particularly at positions farther from the cooling interface.

The β phase, with its BCC crystal structure, was the first to crystallize. Higher cooling rates near the interface led to a greater volume fraction of the β phase in these regions. Consequently, positions near the base experienced higher liquidus isotherm displacement rates and cooling rates, resulting in higher macrohardness values. Lower microhardness near the base is consistent with the crystallization behavior of manganese–aluminum–bronze, where lower temperatures favor the formation of κ intermetallics and reduce the α phase content.

The CuMn11Al8Fe3Ni3 manganese–aluminum–bronze alloy is extensively used in components exposed to harsh environments, such as marine propellers, bearings, and bushings, due to its excellent corrosion resistance and mechanical properties. This study provides critical insights into the relationship between cooling rates during solidification and the resulting microstructural and mechanical properties. Components requiring higher macrohardness, such as marine propellers, benefit from controlled cooling rates that stabilize the β phase and reduce grain size. Conversely, applications like bearings and bushings, which demand enhanced wear resistance and fatigue strength, rely on the precipitation of intermetallic phases, a process facilitated by slower cooling rates. The equations derived in this study enable precise control of cooling parameters, allowing the tailored design of components for specific operational requirements.

This research also demonstrates the potential for optimizing solidification processes to balance productivity and performance. Faster cooling rates, which improve macrohardness and reduce processing time, can be strategically applied to enhance the properties of components where wear resistance is critical. By correlating thermal variables with material performance, this work not only advances the understanding of this alloy but also serves as a guideline for improving the efficiency and reliability of CuMn11Al8Fe3Ni3-based components in industrial applications.

A comparative analysis with similar alloys, such as nickel–aluminum–bronze (NAB) and silicon–aluminum–bronze (SAB), underscores the unique characteristics of CuMn11Al8Fe3Ni3. While NAB and SAB alloys exhibit similar trends in microstructural evolution and hardness variations during unidirectional solidification, the addition of manganese in CuMn11Al8Fe3Ni3 enhances mechanical properties and casting quality. This alloy achieves comparable or superior performance with optimized manganese and iron additions, requiring less nickel than NAB to achieve high hardness and corrosion resistance. These attributes make CuMn11Al8Fe3Ni3 particularly suited for applications demanding a balance of strength, wear resistance, and corrosion performance.

## 5. Conclusions

This study investigated the influence of solidification thermal variables on the microstructure and mechanical properties of the CuMn11Al8Fe3Ni3 manganese–aluminum–bronze alloy. The key findings are summarized as follows:

The unidirectional solidification apparatus was effective in generating a range of cooling rates and distinct microstructures within the alloy. This setup allowed for a controlled study of the correlation between thermal parameters and the alloy’s mechanical properties.

Brinell hardness decreased from 199 HB (13.03 °C/s) near the cooled base to 184 HB (0.23 °C/s) at the top of the ingot, due to grain refinement and stabilization of the β phase at higher cooling rates.

Vickers microhardness increased from 222 HV to 244 HV as the cooling rate decreased, attributed to the higher volume fraction of intermetallic precipitates at slower cooling rates.

The equations presented in this study offer a novel tool to predict and control the effects of cooling rates on microstructure and mechanical properties. These predictive models provide practical insights for optimizing the solidification process, which are not commonly addressed in the literature.

The findings are highly relevant for industrial applications. Faster cooling rates, which enhance Brinell hardness, are ideal for components like marine propellers. In contrast, slower cooling rates, which increase Vickers microhardness, are more suitable for bushings and bearings requiring high wear and fatigue resistance.

As a guideline for future research, the authors suggest performing wear and fatigue tests to evaluate the effects of different cooling rates over time. These tests will enhance the practical applicability of this study, especially in industrial applications requiring excellent wear and fatigue resistance. Additionally, further studies using advanced characterization techniques, such as EBSD and XRD, are planned to deepen the understanding of the relationship between microstructure and mechanical properties.

## Figures and Tables

**Table 1:** Main composition of phases in weight percentage [12].

Phases	Al	Si	Mn	Fe	Ni	Cu
Alpha (α)	12.6	0.4	12.6	2.5	1.3	70.6
Beta (β)	24.6	0.6	13	1	2	58.8
Large dendrite particles	7.2	3.5	27	54.8	1.2	6.3
Small dendrite particles	27.6	5	21.3	39.4	0.9	5.7
Globular precipitates	22.8	1.3	26.6	29.5	3.8	18.4
Cuboid precipitates in α	15.9	1.1	27.5	34.5	2.6	18.4
Small cuboid precipitates in β	23.6	0.4	25.5	32.4	1.2	16.9

**Table 2:** Chemical composition of the supplied CuMn11Al8Fe3Ni3 alloy.

Alloy	Composition (wt%)					
	Cu	Mn	Al	Fe	Ni	Others
CuMn11Al8Fe3Ni3	74.80	11.09	8.96	2.39	1.61	Si—0.09
						Pb—0.03
						Sn—0.04
						Zn—0.86

**Table 3:** Composition after the solidification experiment compared to the reference standard [42].

Alloy	Standard	Composition (wt%)					
		Cu	Mn	Al	Fe	Ni	Others
CuMn11Al8Fe3Ni3	DIN 1982	68–77	8–15	7–9	2–4	1.5–4.5	Si: 0.10 max.
							Pb: 0.05 max.
							Sn: 0.50 max.
							Zn: 1.00 max.
CuMn11Al8Fe3Ni3	After solidification	74.63	11.01	8.93	2.37	1.59	Si: 0.09
							Pb: 0.02
							Sn: 0.04
							Zn: 0.80

**Table 4:** Solidification thermal variables, hardness, microhardness, and phase distribution.

Position (mm)	V_L_ (mm/s)	T_R_ (°C/min)	G_L_ (°C/mm)	Hardness (HB)	Microhardness (HV1)	Phase Distribution β (%)
4	0.46	13.03	28.13	199.1	222.4	32.1
8	0.34	6.90	20.57	197.6	227.6	34.4
12	0.28	2.05	7.37	196.3	228.0	34.8
16	0.24	1.27	5.25	194.2	229.4	35.2
26	0.19	0.38	1.97	190.9	233.2	40.1
35	0.17	0.25	1.67	189.4	234.4	45.3
53	0.14	0.23	1.51	184.3	244.6	48.5

## Data Availability

The original contributions presented in this study are included in the article. Further inquiries can be directed to the corresponding authors.
